# Mapping Hydrophobicity on the Protein Molecular Surface at Atom-Level Resolution

**DOI:** 10.1371/journal.pone.0114042

**Published:** 2014-12-02

**Authors:** Dan V. Nicolau Jr., Ewa Paszek, Florin Fulga, Dan V. Nicolau

**Affiliations:** 1 Department of Integrative Biology, University of California, Berkeley, California, United States of America; 2 Department of Electrical Engineering & Electronics, University of Liverpool, Liverpool, United Kingdom; 3 Department of Bioengineering, McGill University, Montreal, Quebec, Canada; German Research School for Simulation Science, Germany

## Abstract

A precise representation of the spatial distribution of hydrophobicity, hydrophilicity and charges on the molecular surface of proteins is critical for the understanding of the interaction with small molecules and larger systems. The representation of hydrophobicity is rarely done at atom-level, as this property is generally assigned to residues. A new methodology for the derivation of atomic hydrophobicity from any amino acid-based hydrophobicity scale was used to derive 8 sets of atomic hydrophobicities, one of which was used to generate the molecular surfaces for 35 proteins with convex structures, 5 of which, i.e., lysozyme, ribonuclease, hemoglobin, albumin and IgG, have been analyzed in more detail. Sets of the molecular surfaces of the model proteins have been constructed using spherical probes with increasingly large radii, from 1.4 to 20 Å, followed by the quantification of (i) the surface hydrophobicity; (ii) their respective molecular surface areas, i.e., total, hydrophilic and hydrophobic area; and (iii) their relative densities, i.e., divided by the total molecular area; or specific densities, i.e., divided by property-specific area. Compared with the amino acid-based formalism, the atom-level description reveals molecular surfaces which (i) present an approximately two times more hydrophilic areas; with (ii) less extended, but between 2 to 5 times more intense hydrophilic patches; and (iii) 3 to 20 times more extended hydrophobic areas. The hydrophobic areas are also approximately 2 times more hydrophobicity-intense. This, more pronounced “leopard skin”-like, design of the protein molecular surface has been confirmed by comparing the results for a restricted set of homologous proteins, i.e., hemoglobins diverging by only one residue (Trp37). These results suggest that the representation of hydrophobicity on the protein molecular surfaces at atom-level resolution, coupled with the probing of the molecular surface at different geometric resolutions, can capture processes that are otherwise obscured to the amino acid-based formalism.

## Introduction

The shape of, and the physico-chemical properties on the protein molecular surfaces govern the specific molecular interactions in protein-ligand complexes [1]. Therefore, studies as diverse as those on protein folding [2], protein conformational stability [3], inter- and intra- protein interactions [4], molecular recognition [5] and docking [6]; as well as applications-orientated ones, such as drug design [7], [8], protein and peptide solubility [9], crystal packing [10], and enzyme catalysis [11], benefit from an accurate and precise representation of the molecular surfaces. Furthermore, for large, intricate protein complexes, such as ion-channels [12], mechano-sensitive channels [13], or molecular chaperones [14], where the biomolecular functionality occurs on the *inner* molecular surface of the complex, makes the precision of the representation of molecular surfaces even more imperative.

A relatively under-studied aspect of the construction of molecular surfaces is the resolution at which the hydrophobicity is represented. Because the biomolecular recognition is a geometrically-localized and charge- and hydrophobicity-specific event, its accurate description requires the representation of molecular surfaces with the finest resolution possible. However, while the charges are atom-localized and therefore their representation at high spatial resolution is immediate, the assignment of hydrophobicity based on residues inherently translates into its representation at a much lower resolution than that for electrical properties. Several studies [15]–[20] developed “atomic hydrophobicities” proposing different sets of atom types, but a sensitivity analysis regarding the number of atom types, as well as study comparing the protein molecular surfaces obtained using atom- or amino acid-level hydrophobicity is lacking.

Separate from the *physical* resolution of hydrophobicity, i.e., at atom- or amino acid-level, the impact of using different *geometrical* resolutions for the construction of the molecular surface has been also relatively under-studied. Indeed, the representation of the molecular surface, which relies on procedures [21]–[27] that use the protein structure deposited in databases, such as Protein Database, PDB [28], usually uses a geometrical resolution between 1.4 to 5 Å, which represents the size of the small molecular species the proteins interact with. However, as discussed before [29], there are many situations that justify the use of larger probes because the protein interacts with larger objects, e.g., membrane lipid rafts [30], cytoskeleton proteins [31], amyloid plaques [32], biomaterials surface [33], biomedical micro-devices [34], [35] and chromatographic media [36]. Also, from the methodology point of view, the probing of the molecular surfaces with at different geometrical resolutions, i.e., using different probe radii, can reveal structural features of the proteins, e.g., shielding of the hydrophobic core [29].

To this end, the present study proposes a methodology for the derivation of atomic hydrophobicity from any hydrophobicity scale, runs a sensitivity analysis to assess the suitability of alternative atom types, and compares the results obtained with atom- and amino acid-level representation of hydrophobicity on molecular surfaces.

## Methods

### Terminology and definitions

Usually, hydrophobicity defines the property of a physico-chemical unit, i.e., a material, a surface, a molecule, or a chemical group, which reflects a particular density and geometrical distribution of water molecules around that unit. When this property, measured by various methods, reflects the repelling of water molecules, this value, usually negative, is also denominated as hydrophobic. Conversely, when the property reflects an increased density of water molecules around the unit, the measured property, with values usually positive, is denominated as hydrophilicity. A physico-chemical unit, in particular a molecule or a chemical group, could contain various sub-units, e.g., chemical groups, or atoms, respectively, which have distinct and different hydrophobicities and/or hydrophilicities. If at least two, non-contiguous units present a hydrophobic and a hydrophilic character, respectively, the unit is deemed amphiphilic. To avoid confusions resulting from the overlap of terms for different parameters, and for the purposes of the analysis of the characterization of protein molecular surfaces, the following terminology will be used:

- *hydrophobicity* is the measured hydrophobicity of a unit, i.e., atom, or amino-acid, which is hydrophobic and which does *not* have an amphiphilic character, i.e., an atom, or which is *assumed, or assigned* not to have an amphiphilic character, i.e., amino-acids;
- *total hydrophobicity* is the sum of the hydrophobicities of the units, i.e., atoms, or amino-acids, which are exposed on the protein molecular surface, weighted with their respective exposed areas;
- *hydrophilicity* is the measured hydrophobicity of a unit, e.g., amino-acid or atom, which is hydrophilic and which does *not* have an amphiphilic character, i.e., an atom, or which is *assumed, or assigned* not to have an amphiphilic character, i.e., amino-acids;
- *total hydrophilicity* is the sum of the hydrophilicities of the units, i.e., atoms, or amino-acids, which are exposed on the protein molecular surface, weighted with their respective exposed areas;
- *overall hydrophobicity* is the hydrophobicity of the amphiphilic protein (previously [29] denominated as amphiphilicity), calculated as the algebraic sum of the total hydrophobicity and total hydrophilicity of the units exposed on the molecular surface, calculated by either using amino-acid, or atom-based methodologies.

### Proteins

A set of 35 proteins (Table 1) selected from the Protein Bata Bank [28], comprising several representative types, i.e., lactalbumins, lysozymes, ribonucleases, hemoglobins and related proteins, albumins and antibodies, have been selected for the comparison of amino acid- and atom-level representation of amphiphilicity. For the purposes of this contribution the chosen proteins need to have a convex shape. Indeed, the probing of proteins that exhibit concave shapes, most notably channel proteins, by probes with increasing radii will produce unreliable results, because much of their interior molecular surface will be inaccessible to larger probes. Finally, to ensure a representative comparison between the atomic- and amino acid-based hydrophobicity, the selected set of proteins is identical with the one used in a previous contribution [29], which reports on the probing of protein molecular surfaces with probes of different sizes.

**Table 1:** Proteins used for the analysis of molecular surfaces.

Cluster	Protein no.	Protein name	PDB code	Atoms	Residues	Chains
1	1	*α lactalbumin*	1A4V	1092	123	1
2	2	*porcine β-lactoglobulin*	1EXS	1248	160	1
	3	bovine β-lactoglobulin	1BEB	2473	324	2
3	4	**chicken egg-white lysozyme**	1LYZ	1001	129	1
	5	turkey egg-white lysozyme	135L	994	129	1
	6	hen egg-white lysozyme	2LYM	1001	129	1
	7	triciclic lysozyme	2LZT	1001	129	1
	8	mutant phage T4 lysozyme	1L35	1305	164	1
	9	T4 lysozyme	1LYD	1309	164	1
4	10	ribonuclease-A	8RAT	951	124	1
	11	ribonuclease-A	1RBX	956	124	1
	12	bovine ribonuclease-A	3RN3	957	124	1
	13	**ribonuclease-A**	1AFU	1894	248	2
5	14	human oxyhemoglobin	1HHO	2192	287	2
	15	human carbonmonoxy hemoglobin	2HCO	2192	287	2
	16	horse hemoglobin	2DHB	2201	287	2
	17	human hemoglobin A	1BUW	4342	574	4
	18	**human hemoglobin**	1Y4F	4368	574	4
	19	hemoglobin mutant	1A01	4368	574	4
	20	human hemoglobin	1Y4P	4376	574	4
	21	hemoglobin mutant	1A00	4382	574	4
	22	human hemoglobin	1Y46	4382	574	4
	23	human deoxyhemoglobin	2HHB	4384	574	4
	24	human hemoglobin	1Y4G	4366	574	4
	25	hemoglobin mutant	1A0U	4386	574	4
	26	hemoglobin mutant	1A0Z	4386	574	4
	27	recombinant hemoglobin	1C7D	4396	576	3
6	28	human serum albumin complex with octadecanoic acid	1E7I	4496	585	1
	29	recombinant human serum albumin	1UOR	4617	585	1
	30	serum albumin	1E_78	4302	585	1
	31	**human serum albumin**	1AO6	4600	585	1
	32	human serum albumin	1BM0	4600	585	1
7	33	immunoglobulin	1IGY	10002	1294	4
	34	immunoglobulin	1IGT	10196	1316	4
	35	**intact human IgG B12**	1HZH	10355	1344	4

**Note:** The proteins marked in bold are model proteins and those in italics have been also used for the analysis of statistical strength.

The selected proteins have various molecular weights (14 to 148 [kDa]), residues (123 to 1344), isoelectric points (4.5 to 11) and shapes (globular, Y-shaped). Five representative proteins, i.e., lysozyme, ribonuclease, hemoglobin, albumin and IgG (Table 1, in bold) have been selected for an in-depth comparison of the atom-level and amino acid-level representation of hydrophobicities. The full results are presented in the Supporting Information section.

A subset of the hemoglobin class has been selected to test the fine differences between the hydrophobicity represented at atom- and amino acid-level resolution. Briefly, the subset comprises eight mutant structures of the deoxy forms of the protein, with the same number of residues (574), but with (i) the Trp37 residue, i.e., 1A0U and 1A0Z, for the crystal form 1 and 2, respectively; and with residues replacing the Trp37 residue by (ii) Tyr37, i.e., structures 1Y46 and 1A00, for crystal 1 and 2, respectively; (iii) Ala37, i.e., 1Y4F and 1A01, for crystal form 1 and 3, respectively; (iv) Glu37, i.e., 1Y4P, for crystal form 1; and (v) Gly37, i.e., 1Y4G for crystal form 1. A full description of these single residue mutations has been reported elsewhere [37].

### Derivation of atomic hydrophobicities

The atomic amphiphilicities have been calculated as independent variables of the following system of linear equations:

for *j* = 1 to 20; and for each j^th^ amino acid *AA_j_*:
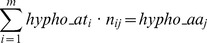
(1)where *j* = amino acid index; *i* = atom type index; *AA_j_* = the j^th^ amino acid; *hypho_at_i_* – atomic hydrophobicity for atom type *i*; *n_ij_* – number of atoms of type *i* in amino acid *j*; *hypho_aa_j_* – hydrophobicity of the amino acid *j*. This system of equations has been solved using several sets of atom types, proposed according to their chemical nature and charge. The number of atom types tested, *m*, was from 8 to 13. The proposed atom type matrices, [n_ij_], are presented in File S1. The system of equations (Eq. 1) has multiple solutions because the number of equations is not equal with the number of variables. For each of these number of atom types the solution retained was the one that presented the best fit, i.e., the smallest standard deviation between the target values (amino acid hydrophobicities) and estimated ones (atomic hydrophobicities multiplied with the n_ij_ respective to amino acid j). The solution that represents the best fit, and consequently the one that has been retained for further calculations contains 12 atom types. The respective matrix, [n_i12_], is presented in Table 2.

**Table 2:** Atom types and relative atomic hydrophobicity (small correlation matrix, see text).

No.	Name	Types	Atomic hydrophobicity [kcal mol^−1^]	
			DGwif	DGoct
1	Cl	Aliphatic C	0.2169	−0.2485
2	Cr	Aromatic C	−0.2607	−0.3332
3	Cx	C bonded to a heteroatom, less O	−0.1217	−0.0217
4	Cox	C bonded to O	−0.2645	0.2613
5	Clp	Aliphatic C – positively charged	1.5299	0.5564
6	Cln	Aliphatic C – negatively charged	−0.9227	−0.9126
7	N	N in amide backbone	−0.0062	−0.0763
8	Np	N – positively charged (amino)	0.3544	0.3748
9	Nl	N in lysine	−0.1231	−0.7263
10	O	O in amide backbone	0.4881	0.9277
11	On	O negatively charged in COOH and OH	0.7653	1.6749
12	S	S in Cys and Met	0.4989	3.029

**Note:** DGwif and DGoct are the free energies of transfer of AcEL-X-LL peptides from water to bilayer, or octanol interface, respectively [38].

### Atom-based hydrophobicities

The initial test of fitness versus the number of atom types, from 8 to 13 atom types, used two hydrophobicity scales, i.e., the hydrophobicity of an amino acid embedded in a penta-peptide [38] as a measure of the enthalpy for its transfer (i) through a lipid membrane (DGwif); and (ii) from water to octanol (DGoct). The results of these calculations are presented in File S2. For the best fit of the atom types (m = 12), additional sets of atomic hydrophobicities have been calculated from other hydrophobicity scales, namely (i) Kyte-Doolittle, KD [39]; (ii) Hopp-Woods, HW [40]; (iii) logP [41] (cf. its implementation in HyperChem); (iv) two “estimated hydrophobic effects”, for “residue burial”, RB; and for “side chain burial”, SCB, [42]; (v) two measurements of HPLC retention, i.e., retn21 and retn74, [43]; (vi) position-specific apparent free energy of membrane insertion, ΔG^app(i)^_app_, at position 0, DGapp_0, [44]; (vii) water-to-bilayer transfer free energy scale, ΔG_sc_^wbi^ [45]; and (viii) unified hydrophobicity scale (UHS) for the water-membrane transfer free energy [46]. The results of these calculations are presented in File S3.

### Molecular surfaces

The molecular surfaces of the selected proteins have been constructed using Connolly’s algorithm [22], [23], which records the position of the points of contact (or at a distance equivalent to the van der Waals radius of the respective atoms) between a virtual rolling probing ball with a set radius and the atoms on the surface of the protein. For amino acid-based overall hydrophobicity, total hydrophobicity and hydrophilicity, their spatial distribution was determined through the allocation, at the point of contact, of the hydrophobicity of the amino acid, weighted by the ratio of the probed surface per the total area of the amino acid. A similar procedure was used for mapping the spatial distribution of the atom-based hydrophobicities. The procedure involved the allocation of specific atomic hydrophobicity weighted with the ratio between the probed atomic area and the total atomic area. The results of the calculations regarding the exposed area vs. probe radii are presented in File S4.

The calculations used an in-house program [29], which is an upgrade of the Connolly’s original software code [23], [47], embedded in a Windows interface. The program has been run on a personal computer with a 64-bit operating system, an Intel Core i7-3630QM CPU @2.40 GHz, and an installed memory of 8GB. The 4D points (x, y, z coordinates and molecular property) have been visualized using DS Viewer Pro. (from Accelerys Inc.). The molecular surfaces have been constructed for all 35 proteins in the dataset (Table 1), for probe radii ranging from 1.4 Å to 20 Å. Beyond probe radii of 20 Å it was found [29] that the change of the properties on the molecular surface is negligible. Consequently the calculations stopped at this threshold.

### Protein properties on the molecular surface

Three types geometrical and physico-chemical properties have been calculated on the molecular surface of the selected proteins: (i) *global properties* (i.e., total surface; overall hydrophobicity, total hydrophobicity and hydrophilicity, for amino acid- and atom-based calculations); (ii) *property relative densities* (i.e., overall and total hydrophobic and hydrophilic relative density, calculated by dividing the property value to the total molecular area); and (iii) *property specific densities* (calculated by dividing the respective property, e.g. total hydrophobicity, to the area that property turns up, e.g. hydrophobic area). For the comparison purposes, the overall hydrophobicity, i.e., the algebraic sum of hydrophobicity expressed in negative numbers; and hydrophilicity expressed in positive numbers, has been calculated for both amino acid-based and atom-based hydrophobicity scales. This methodology, applied here to atom-based properties, was used before [29] but only for amino acid-based properties. The full results are presented in File S5.

The hemoglobins subset has been separately analyzed using the same procedures. To compare the molecular surface properties with the hydrophobicity of the single residue replacement, the values for the proteins that present two crystallographic forms, i.e., 1A0U and 1A0Z for Trp; 1Y46 and 1A00 for Tyr; and 1Y4F and 1A01 for Ala, have been averaged, but those with a single crystallographic form, i.e., 1Y4P for Glu and 1Y4G for Gly, remained unchanged. The full results regarding this subset are presented in File S6.

## Results and discussion

### 1. Atomic hydrophobicity

**Figure 1:**
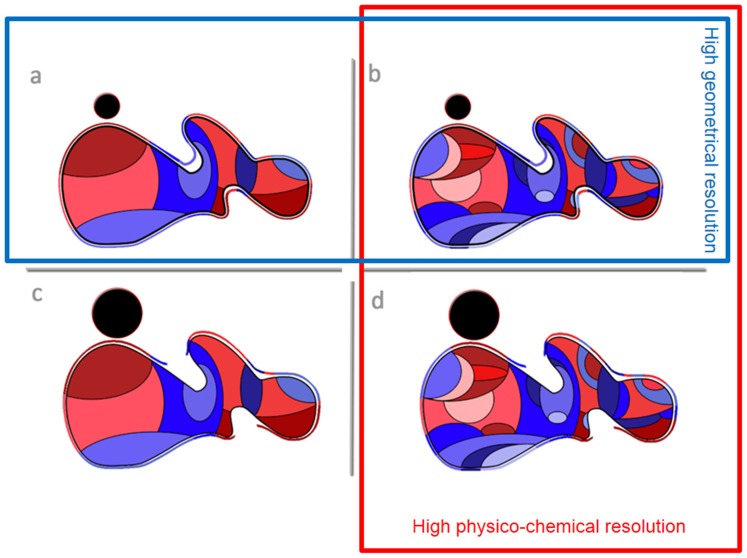
Schematics of different representations of molecular surfaces. Top row: representation of the hydrophobicity on the molecular surface, at (a) low, amino acid-level; and (b) high, atomic-level resolutions. Bottom row, the same representation for molecular surfaces probed with larger probes. Scheme upgraded from [29], which reports the mapping at low, amino acid-based hydrophobicity (i.e., a and c).

Because the charge is an atom-based property, the spatial representation of charges on the protein molecular surface can be inherently performed at atom-level resolution. In contrast, the spatial distribution of hydrophobicity cannot be usually represented at high resolution, because of two reasons. First, as the hydrophobicity is usually assigned to amino acids not to atoms, its spatial representation on the protein molecular surface is constructed at several-atoms resolution, i.e., from patches comprising several atoms belonging to a parent amino acid, which is probed by the molecular surface probing ball. Intuitively, an atom-level representation of hydrophobicity would allow a more precise quantification of the properties manifested on the molecular surface and inference of the molecular recognition between protein and small molecular species. For instance, the role arginine, which comprises chemical groups with various hydrophobicities along the molecule, plays in protein-protein interactions is difficult to be understood within the framework of an evenly distributed hydrophobicity. A schematic of the differences between a molecular surface which is represented at amino acid- and at atomic level is presented in Figure 1, a and b, respectively. Furthermore, constructing the molecular surface using larger probes, which could be relevant to the analysis of the interaction of proteins with larger objects [29], e.g., nanoparticles, flat surfaces, will result in more uncertain quantification of the hydrophobicity, as the molecular surface is represented by a collection of atoms which represent a decreasingly-smaller fraction of their parent amino acids. This situation is presented schematically in Figure 1, c and d, respectively.

Second, in contrast with the charges, hydrophobicities are not represented in a standardized manner, with more than 100 hydrophobicity scales being presently proposed. Although “hydrophobic potentials” have been proposed [48]–[51], including some for atomic level representations [41], [50]–[53], the non-standardized hydrophobicity, in particular at atom level, precludes their universal use.

There are several possible avenues for the derivation of atomic hydrophobicities, either independent of the Accessible Solvent Area (ASA), as used primarily in this work; or accounting for ASA when solving the system of equations (Eq. 1). Probing the molecular surface at different geometrical resolutions –a central methodological tool for assessing the structuring of the molecular surface, will result in different ASA’s for different probe radii (see File S7). Consequently, if ASA’s are used as weighting factors for the calculation of atomic hydrophobicities, then the solution of the system of equations (Eq. 1) will be dependent on the radius of the probe used for the construction of the molecular surface. Equally important, the equivalence between atomic hydrophobicities and amino acid ones from which they are derived will cease, thus making the comparison between the two methods of constructing the distribution of hydrophobicity and hydrophilicity on the protein molecular surface inoperable. Furthermore, if ASA’s are used for the calculation of atomic hydrophobicities, their equivalent formalism with atomic charges also cease to exist, making their possible use for the development of hydrophobic potential also inoperable. A full treatment of the modes of calculation of atomic hydrophobicities is presented in File S8. For all these reasons, and although we report results obtained both accounting or not ASA’s (see File S3 and S4), the further analysis will mainly use the atomic hydrophobicities obtained from Eq. 1.

### 2. Derivation and use of atomic hydrophobicities

While several atomic hydrophobicity scales have been proposed in the last decades, they present several limitations. For example they (i) are estimated from large QSAR databases where amino acids represent a small fraction of the archived molecules [54], [55], thus skewing the results away from the residues of interest for the analysis of proteins; or (ii) propose a small number of atom types, e.g., m = 5 [15], [16], [56], m = 6,7 [18], [20], m = 8 [17], thus potentially not being able to describe the molecular surface with sufficient atom-specificity; or (iii) use proprietary parameters [41], [57]; (iv) use “hydrophobic potentials” (the analogue to electrostatic potentials), usually embedded in proprietary software [41], [58]–[60]; or (v) result from the compilation of several different sources [61], [62]. Most importantly, none of these atom-based hydrophobicity scales are derived from amino acid-based ones, therefore making the comparison of molecular surfaces constructed using amino acid-, or atom-based hydrophobicities difficult. The methodology for the derivation of atom-based hydrophobicity proposed here attempts to address many of these limitations.

Several sets of atomic hydrophobicities are proposed, each calculated for a number of representative atom types, varying from 8 atom types, i.e., starting with the set proposed by Efremov at al. [17], to 14. The selection of the atom types was based on the chemical structure and environment of the respective amino acid. For m = 8 the atom types are: Cl – aliphatic C; Cr – aromatic carbon; Cx – carbon linked to a heteroatom; N – uncharged nitrogen; O – uncharged oxygen; S – sulphur; Np – positive charged nitrogen; and On – negatively charged oxygen. For m = 12 this set was expanded by splitting the C atoms types according to their charge, i.e., in conformity with the charges assigned by the Amber force field [63]; and creating a new atom type for the N atom in lysine. The representative atom types for m = 12 are presented in Table 2.

The criterion for the choosing the optimum number of atom types has been the overall (i.e., for all 20 amino acids) best fit of the *estimated* atom-based hydrophobicities compared to the *actual* amino acid-based ones used for calculations. Two hydrophobicity scales, i.e., the hydrophobicity of an amino acid embedded in a penta-peptide, [38] derived from the thermodynamic measurements of the enthalpy of the transfer of the respective peptide through a lipid membrane (DGwif); and from water to octanol (DGoct), respectively, have been used to calculate the best fit between atom-based and amino acid based hydrophobicities. The best fit increased moderately, but steadily, with the increase of the number of atom types, m, from 8 to 12. For m = 13 the improvement of the fit ceased and for m = 14 the system could not be solved anymore. The detailed discussion on these results is presented in File S8 and a full description of the data is presented in Files S1–S5. The evolution of the fit with the number of atom types is presented in Table 3.

**Table 3:** Fit for 12 atom types for different hydrophobicity scales.

Scale	R^2^	Standard deviation
DGwif	0.95	0.86
DGoct	0.97	1.64
DGhx	0.97	8.51
DGsa	0.82	5.5
KD	0.89	2.79
WD	0.95	1.88
Log P	0.96	1.61
DGaa_0	0.87	0.28
DGsc_wbi	0.97	0.36
DGwm_UHS	0.82	0.18

**Note:** DGwif, DGoct, DGhx and DGsa are the free energies of transfer of AcEL-X-LL peptides from water to bilayer, or octanol interface, respectively [38]; Kyte-Doolittle, KD [39]; Hopp-Woods, HW [68]; partition coefficient, logP [41]; position-specific apparent free energy of membrane insertion at position 0, DGapp_0 [44]; water-to-bilayer transfer free energy scale, DGsc_wbi [45]; and unified hydrophobicity scale (UHS) for the water-membrane transfer free energy, DGwm_UHS [46].

### 3. Protein overall hydrophobicity on the molecular surface

Once the optimum set of atom-based hydrophobicity, i.e., atom types and the values of the atomic hydrophobicities, has been established, one can quantify the protein overall hydrophobicity manifested on its molecular surface, and compare it with the one calculated with the classical amino acid-based hydrophobicity. The following discussion will focus on five representative proteins, i.e., lysozyme, ribonuclease, hemoglobin, albumin and IgG, which have vastly different molecular weights, i.e., from 129 to 1344 residues (Table 1, in bold); and shapes, i.e., globular, ellipsoidal and Y-shaped. While the following results are discussed for the DGwif-derived hydrophobicity only, similar results are obtained for all other hydrophobicity scales. The full results for all 35 model proteins are presented in File S5.

**Figure 2:**
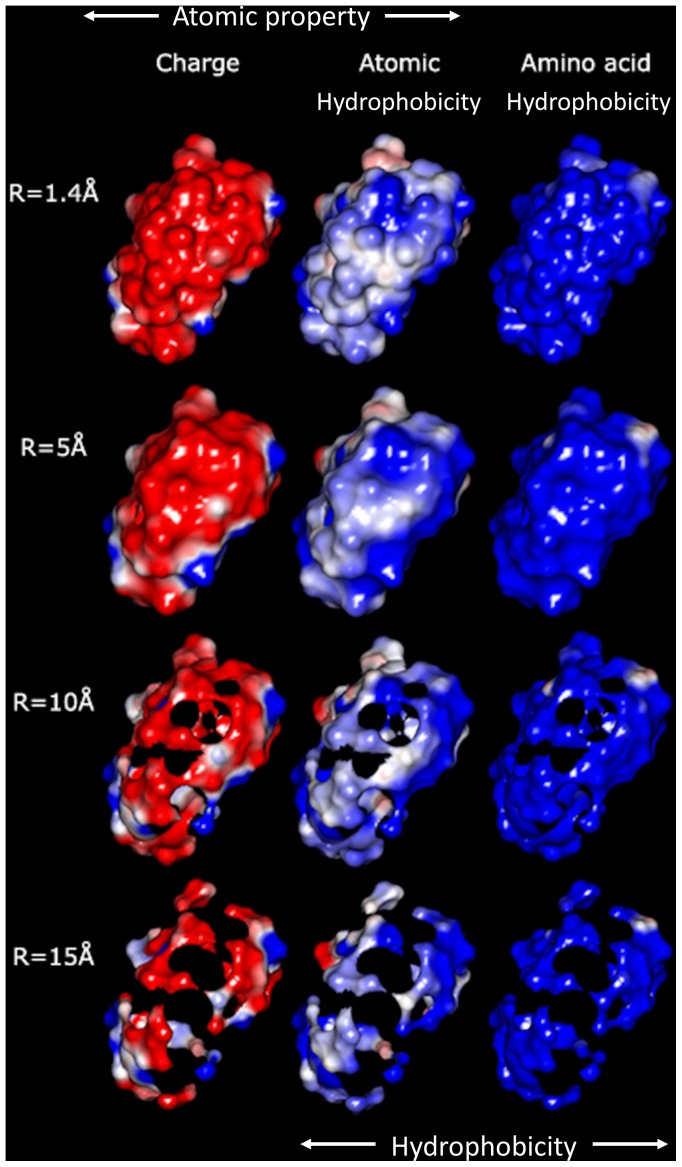
Comparison between the representation of atom-based properties, i.e., charges (left column; red = negative, blue = positive), atomic hydrophobicity (middle column; red = hydrophobic and blue = hydrophilic region); with amino acid-based properties, i.e., amino acid-based hydrophobicity (right column) on the molecular surface of ribonuclease (PDB ID: 1AFU). The molecular surface is probed with decreasing geometrical resolution (from top to bottom).

**Figure 3:**
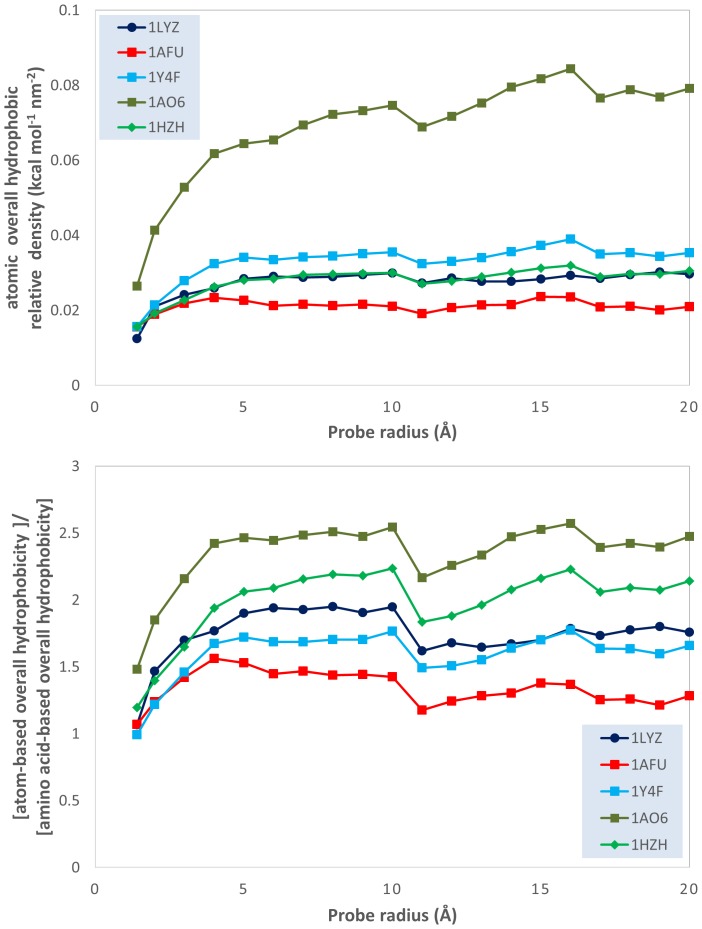
Evolution of the ratio between atom-based overall hydrophobicity and total molecular surface area (relative density of the atomic hydrophobicity); and of the ratio of the atomic and the amino acid overall hydrophobicities; vs. probe radii for 5 model proteins: lysozyme (1LYZ); ribonuclease-A (1AFU); human hemoglobin (1Y4F); human serum albumin (1AO6); human IgG (1HZH).

The comparison of the molecular surface (Figure 2 for ribonuclease) allows a qualitative discrimination between properties calculated at atom-level resolution, but of a different nature, i.e., charges and atomic hydrophobicity (Figure 2, left and middle columns, respectively); as well as between those of the same nature, i.e., hydrophobicity, but calculated at atom- and amino acid-level (Figure 2, middle and right columns, respectively). A preliminary inspection shows that the distribution of atomic hydrophobicity, despite being physico-chemically similar with the amino acid hydrophobicity, from which it is actually derived, resembles far more the distribution of charges on the molecular surface. Indeed, the molecular surface represented by amino acid hydrophobicity remains largely, and evenly, hydrophilic, regardless of the geometrical resolution it is probed at. Conversely, the molecular surface represented by atomic hydrophobicity offers a far more varied landscape. For instance, several hydrophobic ‘fingers’, not detected by the amino acid hydrophobicity molecular surface, but visible as near-zero charges on the charge molecular surface (Figure 3, left column), remain apparent, regardless of the probe radii. A more detailed graphical representation of the evolution of the property-molecular surface is presented in File S9.

#### Atomic and amino acid based hydrophobicities

**Figure 4:**
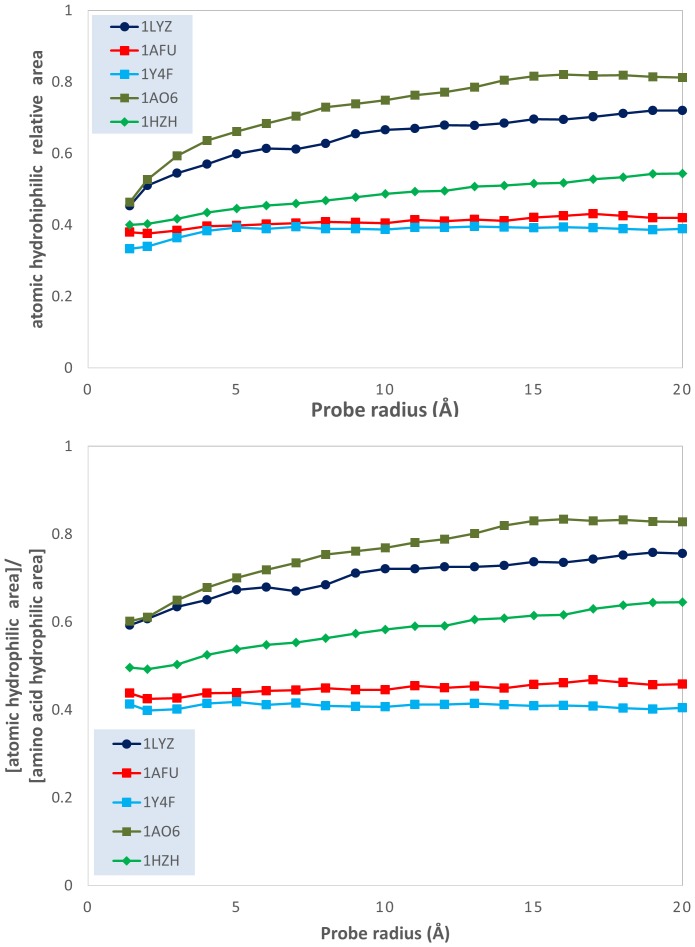
Evolution of the ratio between the atomic hydrophilic area and the total molecular surface area; and of the ratio between the atomic and the amino acid hydrophilic areas; vs. probe dimensions for 5 model proteins: lysozyme (1LYZ); ribonuclease-A (1AFU); human hemoglobin (1Y4F); human serum albumin (1AO6); human IgG (1HZH).

**Figure 5:**
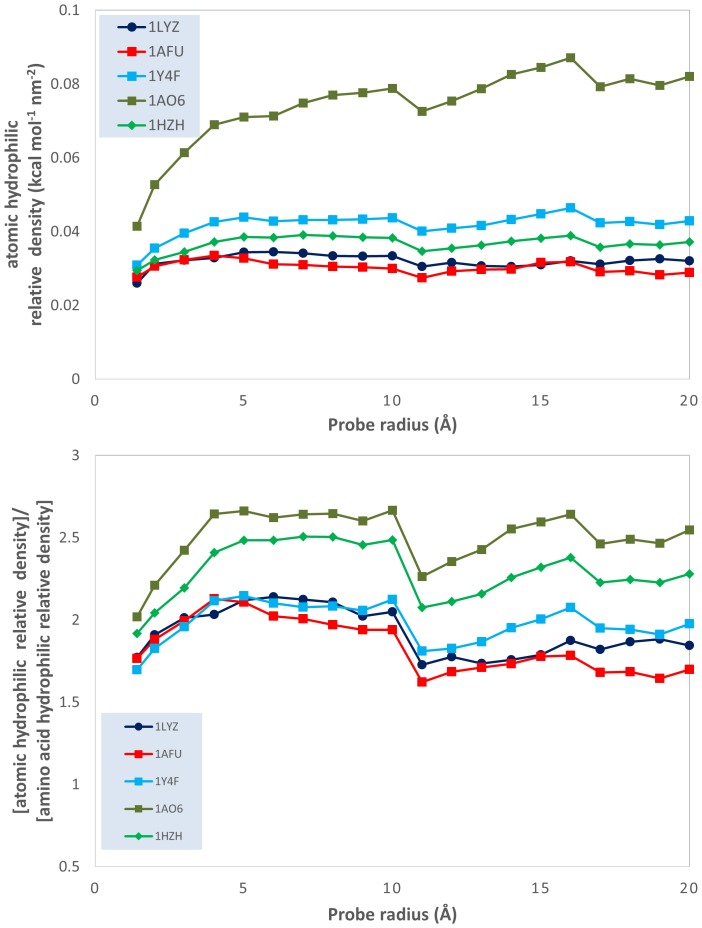
Evolution of the ratio between the atomic hydrophilicity and the total molecular surface area (hydrophilic *relative* density); and of the ratio between the atomic and the amino acid hydrophilicity relative densities; vs. probe dimensions for 5 model proteins: lysozyme (1LYZ); ribonuclease-A (1AFU); human hemoglobin (1Y4F); human serum albumin (1AO6); human IgG (1HZH).

**Figure 6:**
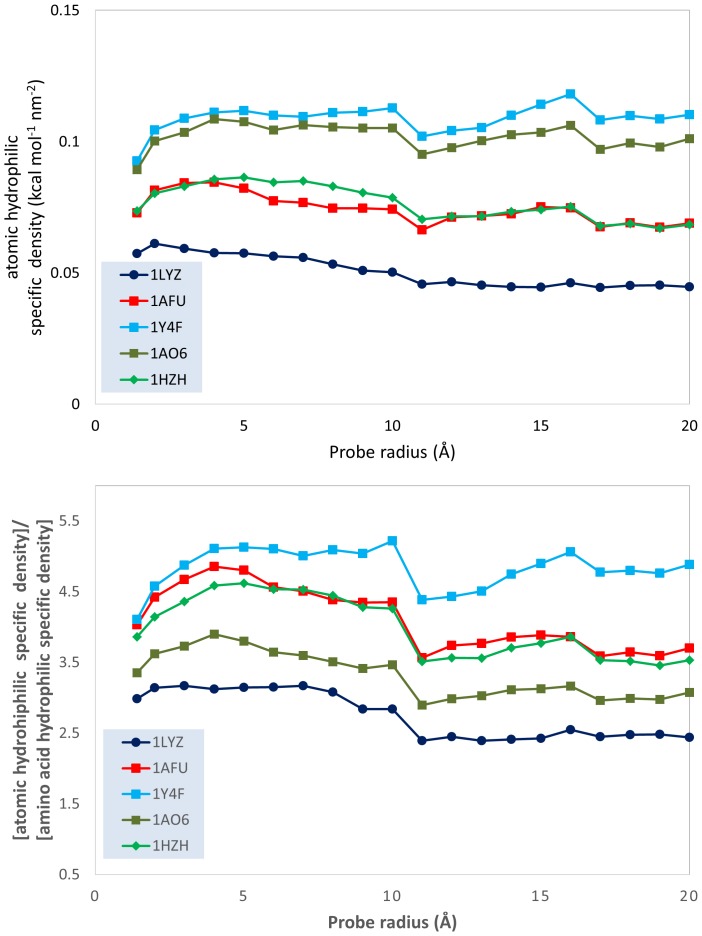
Evolution of the ratio between the atomic hydrophilicity and the hydrophilic area (hydrophilic *specific* density); and of the ratio between the atomic and the amino acid hydrophilicity specific densities; vs. probe dimensions for 5 model proteins: lysozyme (1LYZ); ribonuclease-A (1AFU); human hemoglobin (1Y4F); human serum albumin (1AO6); human IgG (1HZH).

**Figure 7:**
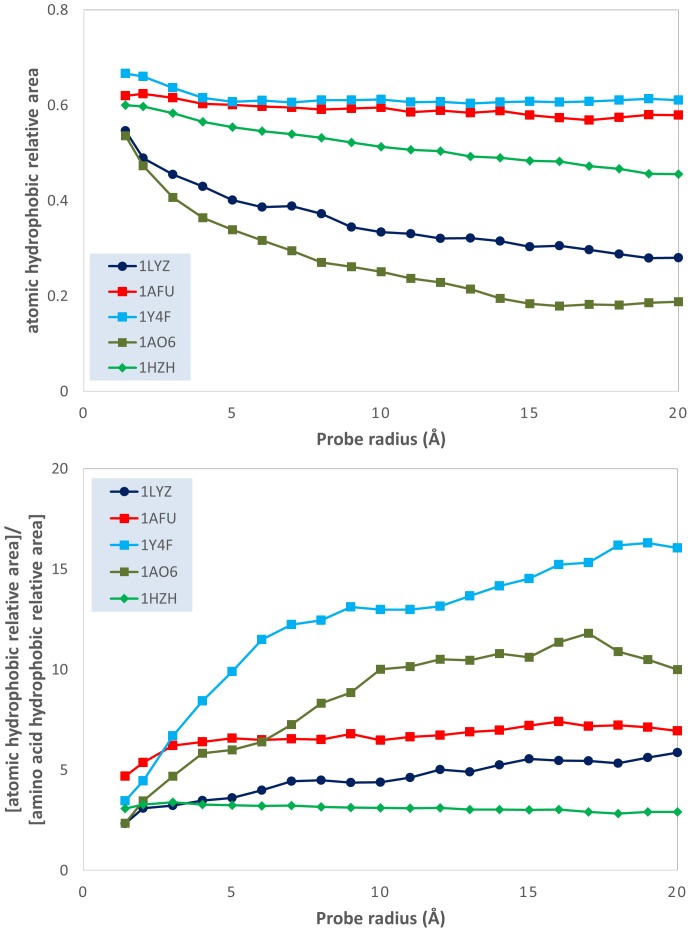
Evolution of the ratio between the atomic hydrophobic area and the total molecular surface area; and of the ratio between the atomic and the amino acid hydrophobic relative areas; vs. probe dimensions for 5 model proteins: lysozyme (1LYZ); ribonuclease-A (1AFU); human hemoglobin (1Y4F); human serum albumin (1AO6); human IgG (1HZH).

**Figure 8:**
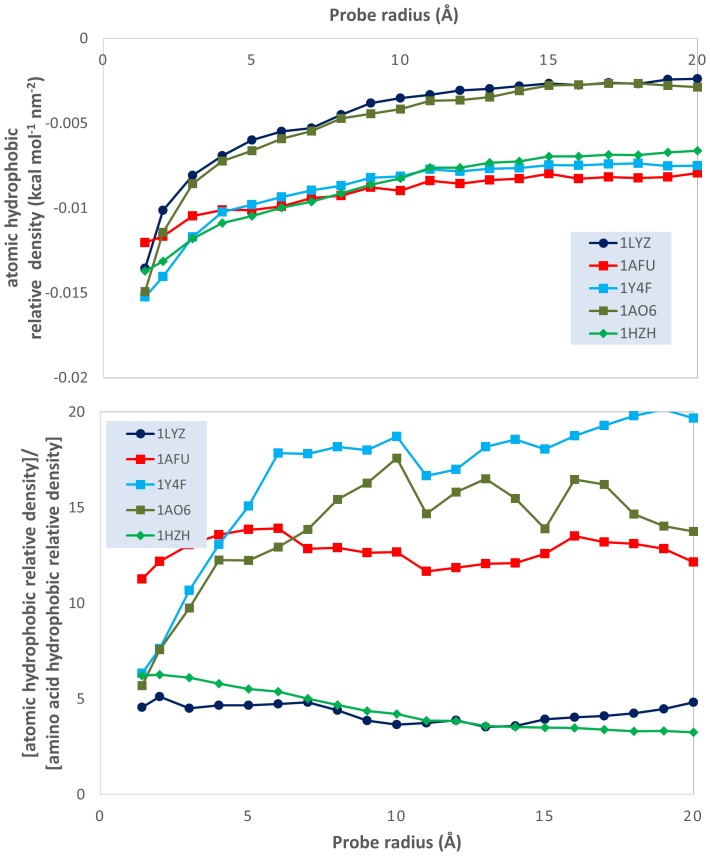
Evolution of the ratio between the atomic hydrophobicity and the total molecular surface area (hydrophobic *relative* density); and of the ratio between the atomic and the amino acid hydrophobicity relative densities; vs. probe dimensions for 5 model proteins: lysozyme (1LYZ); ribonuclease-A (1AFU); human hemoglobin (1Y4F); human serum albumin (1AO6); human IgG (1HZH).

**Figure 9:**
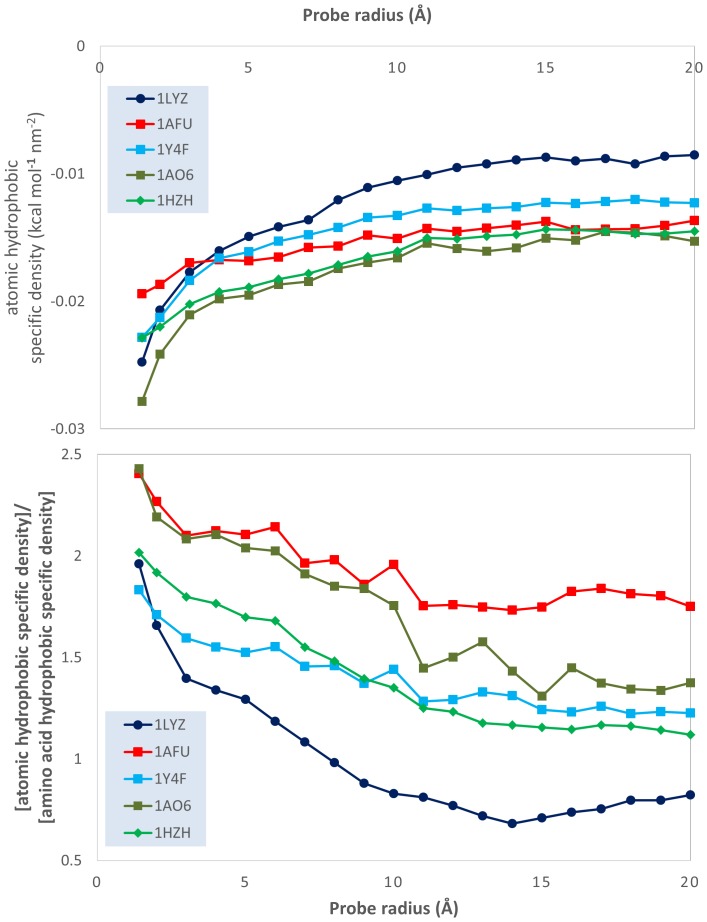
Evolution of the ratio between the atomic hydrophobicity and the hydrophobic area (hydrophobic *specific* density); and of the ratio between the atomic and the amino acid hydrophobicity specific densities; vs. probe dimensions for 5 model proteins: lysozyme (1LYZ); ribonuclease-A (1AFU); human hemoglobin (1Y4F); human serum albumin (1AO6); human IgG (1HZH).

This qualitative analysis is also supported by quantitative data, which could also provide a more detailed physical insight. The variation of the atomic physico-chemical properties, i.e., overall hydrophobicity, total hydrophilicity and hydrophilicity, as well as their derived measures, i.e., relative area (hydrophilic or hydrophobic area divided by total molecular surface area), relative density (overall hydrophobicity, total hydrophobicity or hydrophilicity divided by the total molecular surface area) and specific density (hydrophilicity or hydrophobicity divided by their respective area) with the variation of the probe radius is presented in Figures 3–9 (top panels); and a synthetic overview of these parameters is presented in Table 4. Table 4 also presents the comparison between the atomic and their homologue amino acid properties (also presented in Figures 3–9, bottom panels).

**Table 4:** General comparison of the evolution of molecular surface properties with the probe radius, calculated at atom- and amino acid level.

Property (definition)	Atomic property relationship vs. probe radius (R) increase	Atomic property ratio to amino acid homologue
**Overall hydrophobicity**		
**Relative density^1,2^**: Overall hydrophobicity/Molecular surface area	Slight increase for most proteins Large increase (from ∼0.02 to ∼0.08 kcal nm^−2^) for 1AO6**^3^** [Figure 3 top]	Generally larger (up to 2.5x); Generally increase for R = 1 to 5 Å, then constant [Figure 3 bottom]
**Hydrophilicity**		
**Relative area**: Hydrophilic area Total molecular area	Constant, i.e., ∼40% of the total area (1AFU, 1Y4F) or increase from 40% to 50% (1Y4F), 60% (1LYZ) and 80% (1AO6) [Figure 4 top]	Generally smaller, between 40% to 80% Constant (40%, 1Y4F, 1AFU), or increase from 50% up to 80% [Figure 4 bottom]
**Relative density**: Total hydrophilicity Molecular surface area	Slight increase for most proteins Large increase (from ∼0.02 to ∼0.08 kcal nm^−2^) for 1AO6 [Figure 5 top]	Generally larger –1.5 to 2.5x Generally constant with R [Figure 5 bottom]
**Specific density**: Total hydrophilicity Hydrophilic area	Rather constant or a slight decrease (1LYZ) [Figure 6 top]	Much larger, i.e., 2.5–5.5x; Slight decrease with R [Figure 6 bottom]
**Hydrophobicity**		
**Relative area**: Hydrophobic area Total molecular area	Constant, i.e., ∼40% of the total area (1AFU, 1Y4F) or decrease from 60% to 50% (1Y4F), 40% (1LYZ) and 20% (1AO6) [Figure 7 top]	Much larger, between 2.5x to 17x Constant (1HZH) or increase with R [Figure 7 bottom]
**Relative density**: Total hydrophobicity Molecular surface area	Slight decrease**^4^** from −0.015 to −0.01 kcal nm^−2^ (1HZH, 1AFU, 1Y4F). Large decrease, from ∼0.015 to ∼0.005 kcal nm^−2^ (1AO6, 1LYZ) [Figure 8 top]	Much larger 5 to 20x Generally constant with R [Figure 8 bottom]
**Specific density**: Total hydrophobicity Hydrophobic area	Decrease from −0.03 and 0.02 to −0.02 and −0.01 kcal nm^−2^ [Figure 9 top]	Generally larger, i.e., 1–2.5x;Decrease with R [Figure 9 bottom]

**Notes:**

1. Overall hydrophobicity is the algebraic sum of hydrophilicity (positive sign) and hydrophobicity (negative sign). Consequently, the increase of the overall hydrophobicity means that it is more hydrophilic.

2. The relative density of the overall hydrophobicity is equal to its specific density.

3. PDB codes for model proteins: lysozyme (1LYZ); ribonuclease-A (1AFU); human hemoglobin (1Y4F); human serum albumin (1AO6); human IgG (1HZH).

4. Hydrophobicity is expressed in negative numbers. Consequently, a decrease in hydrophobicity will be represented by a move towards 0.

The qualitative (Figure 2) and quantitative data (Figures 3–9 and Table 4) allows for the construction of the following framework regarding the structuring of the protein molecular surfaces:

- *Overall hydrophobicity.* The slight, or –for albumin- considerable, increase of the *density of overall hydrophobicity* with the probe radius (Figure 3, top) indicates that protein molecular surfaces are more hydrophilic towards their outer edges, which is consistent with the “hydrophobic core” model. Moreover, the considerable (approximately two times) higher values obtained for atom-level density of overall hydrophobicity compared with amino acid ones (Figure 3, bottom) suggest that amino acid-based formalism underestimates the “hydrophobic core” structuring of the molecular surface.
- *Total hydrophilicity*. The slight increase of the *atomic hydrophilic relative area* with the probe radius (Figure 4, top) and the slight-to-considerable increase of the *atomic hydrophilic relative density* with the probe radius (Figure 5, top) also supports the “hydrophobic core” model. However, this observation needs to be qualified: the atom-based calculations reveal *lower* hydrophilic areas (Figure 4, bottom) and *higher* hydrophilic relative densities (Figure 5, bottom) than the homologue values obtained by amino acid-based calculations. This apparent contradiction can be reconciled if we assume that the hydrophilic areas are more “hydrophilicity intense” than predicted by amino acid calculations. The much higher atomic *hydrophilic specific density* than its amino acid counterpart (Figure 6, bottom) also supports this interpretation.
- *Total hydrophobicity*. The above conclusion is also supported by hydrophobicity calculations. Indeed, the slight-to-considerable decrease of the *hydrophobic relative area* with the probe radius (Figure 7, top) and the considerable decrease of the hydrophobic relative density (Figure 8, top) support the “hydrophobic core” model. However, the much larger prediction of the hydrophobic areas by atomic based calculations compared with amino acid ones (approximately 5 times even for the smallest radius considered, but above 10–15 times for some proteins (Figure 7, bottom) suggests a much larger extent of the hydrophobic molecular surface predicted by atom-based calculations than amino acid ones. Apparently, the “hydrophobic intensity” of these extended hydrophobic areas is also considerably higher (Figure 8, bottom) than those calculated from amino acid properties. The higher, but decreasing with the probe radius, ratio between the atomic *hydrophobic specific density* and its amino acid counterpart (Figure 9, bottom) results from the coupling of the decrease of the former (Figure 9, top) and the constant values for the latter [29].
- *Atom-based description of protein molecular surfaces.* The observation that the atom-based representation of the molecular surfaces has considerably higher resolution, coupled with the fact that the respective atomic hydrophobicities have been derived directly from a chosen amino acid hydrophobicity scale, leads to the description of the protein molecular surface with better accuracy and precision than that using amino acid hydrophobicities. While both atom- and amino acid-based calculations describe the protein molecular surface as hydrophilic, and more so with the increase of the probe radius, the atom-level description reveals a “leopard skin” design, with more intense hydrophobic and hydrophilic patches than the rather uniform-hydrophilic surface predicted by the amino acid calculations. Moreover, considering the *specific hydrophobic density,* the validity of the hydrophobic core concept appears not to be fully supported by amino acid-based calculations, especially for large proteins (where it should be the most apparent, [64], [65]), but it is valid if atom-based hydrophobicity is used. These observations lead to the conclusion that atom-based hydrophobicities offer a better representation of the protein molecular surface, as demonstrated by the general agreement with the “hydrophobic core” concept. The molecular surfaces depicted in Figure 2 support these conclusions.

### 4. Analysis of a homologous set of proteins

**Figure 10:**
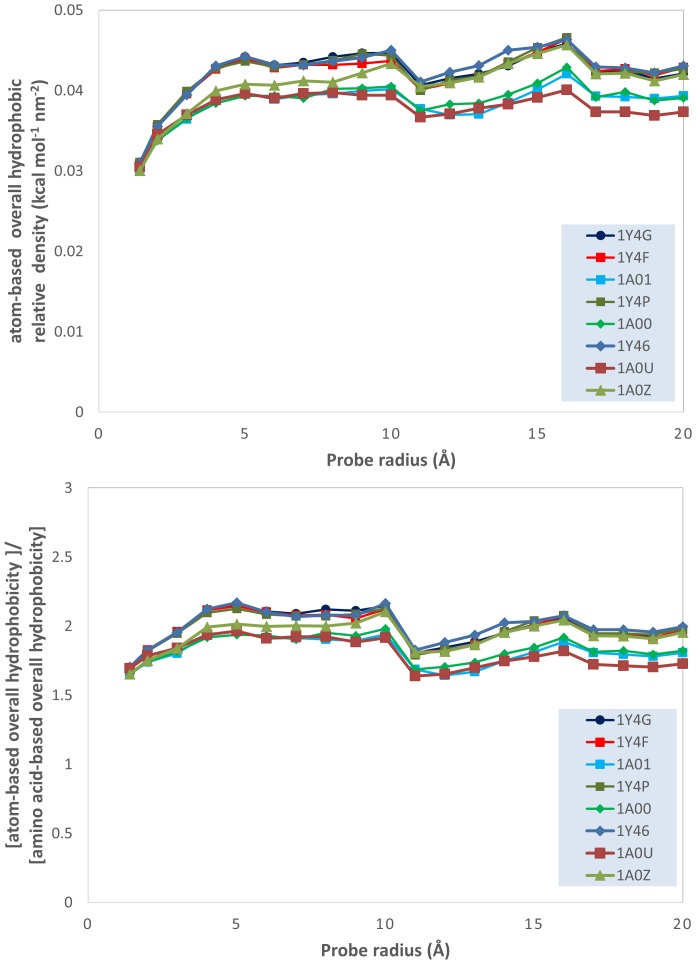
Evolution of the ratio between atom-based overall hydrophobicity and total molecular surface area (relative density of the atomic overall hydrophobicity); and of the ratio of the atomic and the amino acid overall hydrophobicities; vs. probe dimensions for hemoglobin subset.

A more precise comparison of the differences between the atom- and amino acid-based hydrophobicity quantified on the protein molecular surfaces is occasioned by the analysis of a sub-set of hemoglobin single-residue mutants. Because the proteins in this sub-dataset are much more similar between themselves than the rest of the proteins in the overall, larger data set, as only one residue (Trp37) is different, and because this replacement, with Ala, Gly, Glu and Tyr, did not lead to substantial changes in the tertiary structure of the hemoglobins [37], the evolution of the molecular surface parameters with the probe radius is expected to be much closer than that for very different proteins. While this assumption is qualifiedly true, all conclusions drawn from the analysis of very different proteins, as described in the above section, are validated by the analysis on the hemoglobin dataset (see File S6). For example, the evolution of the density of the overall hydrophobicity with the radius of the probe (Figure 10), reveals an increasingly hydrophilic surface with the decrease of the probing resolution; and a higher hydrophilicity (approximately two times) of the molecular surface than that predicted by the amino acid calculations.

**Figure 11:**
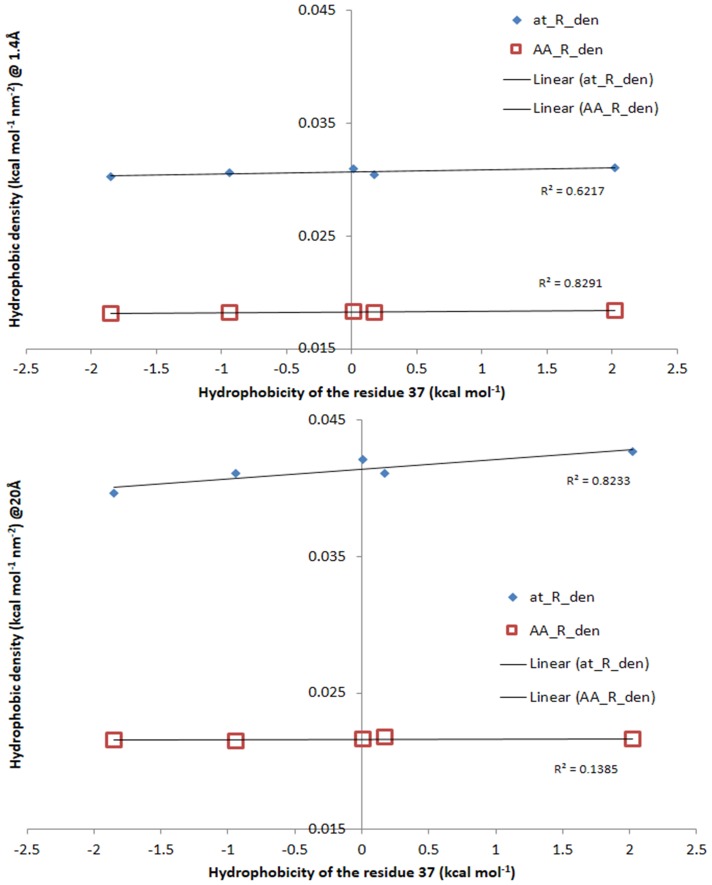
Density of the overall hydrophobicity on the molecular surface of hemoglobin subset, at small (top) and large (bottom) probe radius vs. the hydrophobicity of the residue 37. The hydrophobicities of the residue 37 are, from left to right, Gly, Ala, Glu, Tyr and Trp [38].

Working with very similar set of proteins could lead to important conclusions following the removal of the “noise” caused by too large variations. For instance, the amino acid-based overall hydrophobicity density is essentially identical for all hemoglobins, for both the finest and the coarsest probe, i.e., 1.4 Å and 20 Å, respectively (Figure 11, top and bottom, respectively). However, while the density of the atomic hydrophobicity for the finest probing resolution is also identical for all hemoglobins (albeit larger than amino acid homologue), the calculations for the coarsest probing shows an overall hydrophobicity density that seems to be protein-specific and correlated with the hydrophobicity of the amino acid that replaced the Trp37 in the natural hemoglobin structure.

### 5. Computing time

For the computing system used in this study, the run time ranges from 2 sec for a small protein (lysozyme, 1LYZ, 1001 atoms) for the smallest probe radius (1.4 Å); to nearly 5000 sec for a large protein (IgG, 1HZH, 10196 atoms) for the largest probe radius considered (20 Å), as presented in Table 5. No difference has been noted between the calculations using amino acid hydrophobicities and those using atomic ones.

**Table 5:** Computing time (sec) for the construction of protein molecular surfaces on a personal computer.

Protein (PDB Id)	1LYZ	1AFU	1Y4F	1AO6	1HZH
**No. atoms→ Probe radius↓**	1001	1894	4368	4600	10355
1.4	2	4	6	8	22
5	12	30	96	110	224
10	27	81	777	853	1447
15	33	105	1470	1678	3170
20	48	120	1722	1865	4939

### 6. Perspectives and future directions of research

The present study has demonstrated the benefits of using finer scale, atom-level description of hydrophobicity. These benefits could be further amplified pursuing several possible future directions of research:

- *Molecular surface databases*. A recent comprehensive review of the present understanding of hydrophobicity [66], suggested that it would be beneficial to archive the data regarding the distribution of hydrophobicity and hydrophilicity on the molecular surface of the proteins, in particular those that have the structures deposited in the PDB. It was also suggested that this desideratum can be achieved through molecular simulations from which the fluctuations of the density of water molecules can be calculated. While this research avenue is certainly desirable, the calculations could be expensive and time consuming, even with the emergence of more powerful supercomputers. An interim solution could be the mapping of protein surfaces using atomic hydrophobicities, either the ones reported here, or others calculated using similar methodologies. Furthermore, once the atomic hydrophobicities of interest are derived, one can attempt to cluster the molecular surfaces of whole or parts of proteins through the comparison of atomic neighborhoods, as proposed recently [67].
- *Universality of atomic hydrophobicities*. The present study described how atomic hydrophobicities can be derived from amino acid ones. While different niche applications would find a particular hydrophobicity scale more relevant than another, e.g., chromatography vs. lipid membranes, a standardization of atomic hydrophobicity would greatly help the transfer of knowledge from one application to another. This desideratum can be achieved via two approaches. First, one approach could consist in assigning atom types in accordance to wide-spread used force field, e.g., AMBER. This approach would have the benefit of creating ‘hydrophobic charges,’ which can then be easily used in molecular surface representations, including the calculation of ‘hydrophobic potentials’, such as those previously proposed [41]. Second, a more thorough, albeit computational intensive, approach would be to derive the atomic hydrophobicities from molecular dynamics simulations, e.g., distribution of water molecules around particular atoms, quantification of the fluctuations of water molecules distribution, as alluded above, etc. Aside from the large effort required, this approach would have the benefit of creating truly universal atomic hydrophobicities, as the procedure could be applied to any molecules, e.g., DNA, ligands, glycopeptides, etc. thus opening new avenues for fundamental studies in molecular biology or for applied research, such as drug discovery.

## Conclusion

The mapping and quantification of the physico-chemical properties on the molecular surfaces of proteins using atomic hydrophobicities derived from the corresponding amino acid hydrophobicities scales, offers insights into the structuring of the protein molecular surfaces. The demonstration of the finer representation of protein molecular surfaces at atom level justifies the derivation of sets of these hydrophobicities for any chosen hydrophobicity scale that is appropriate for a specific application, thus opening the opportunity for the engineering of optimum protein-small ligand interactions, as well as protein-solid surfaces interactions. Furthermore, the results are expected to benefit both fundamental studies of protein function and drug discovery by providing a pathway for high resolution mapping of hydrophobicities on the molecular surface.

## Supporting Information

File S1 **Construction of various sets of atom types, from M = 8 to M = 13.**
(XLSX)

File S2 **Selection of the best atom types set by the regression of various sets of atom types (M = 8 to 12) for the hydrophobicity scale proposed by Wimley & White** [38] **.**
(XLSX)

File S3 **Calculation of the best atomic hydrophobicity sets for M = 12 and for various hydrophobicity scales when ASA is considered (Part 1) and when it is not considered (Part 2).**
(XLSX)

File S4 **Calculation of the Accessible Solvent Areas (ASA) for each atom in each amino acid as a function of the probe radius.**
(XLSX)

File S5 **Complete set of data regarding the calculation of physico-chemical properties on the molecular surface of the proteins in the total set (** **Table 1** **), for atomic, amino acid and charges, the latter two from** [29] **.**
(XLSX)

File S6 **Complete set of data regarding the calculation of physico-chemical properties on the molecular surface of the proteins in the selected set of hemoglobins, for atomic, amino acid and charges.**
(XLSX)

File S7 **Example of molecular surface obtained by probing the protein with a small and a large probe.**
(TIF)

File S8 **Comprehensive discussion regarding the possibilities of calculation of atomic hydrophobicities.**
(DOC)

File S9 **Molecular surfaces of ribonuclease presented as a function of the probing resolution, from the finest (top) to the coarsest (bottom).** The molecular surfaces are represented for charges (left column); amino acid-based hydrophobicity (right column); and atom-based hydrophobicity (middle columns). The atom-based molecular surfaces are presented using values directly derived from (Eq.1) – left middle column; and normalized to fit the range of the amino acid hydrophilicities – right middle column.
(TIF)
