# Supplementary figures and images for: Mapping Hydrophobicity on the Protein Molecular Surface at Atom-Level Resolution
Source: PLoS One. 2014 Dec 2;9(12):e114042. doi: 10.1371/journal.pone.0114042 (PMC4252106; doi:10.1371/journal.pone.0114042)

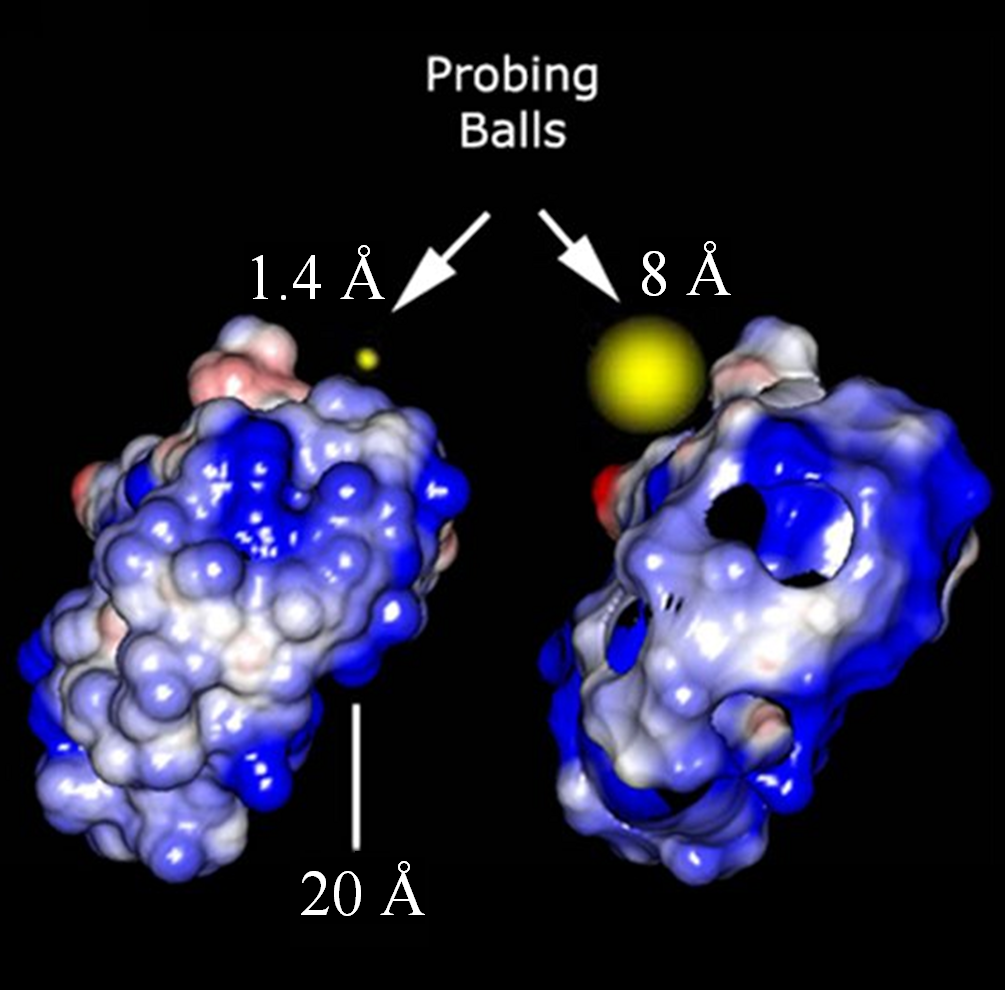

Supplement: File S7 — Example of molecular surface obtained by probing the protein with a small and a large probe. (TIF) [file pone.0114042.s007.tif]

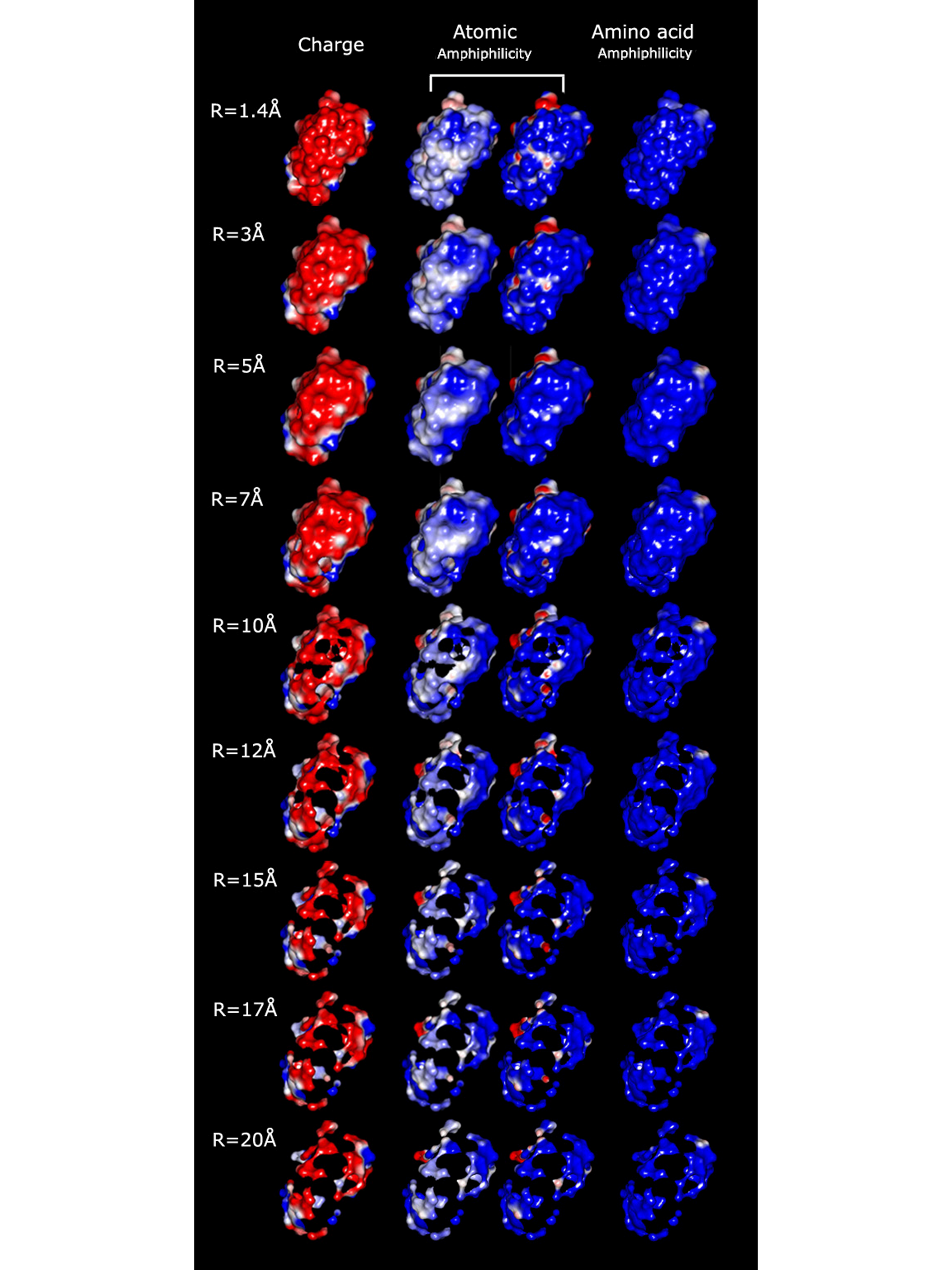

Supplement: File S9 — Molecular surfaces of ribonuclease presented as a function of the probing resolution, from the finest (top) to the coarsest (bottom). The molecular surfaces are represented for charges (left column); amino acid-based hydrophobicity (right column); and atom-based hydrophobicity (middle columns). The atom-based molecular surfaces are presented using values directly derived from (Eq.1) – left middle column; and normalized to fit the range of the amino acid hydrophilicities – right middle column. (TIF) [file pone.0114042.s009.tif]
