# Supplementary material for: Mapping Hydrophobicity on the Protein Molecular Surface at Atom-Level Resolution
Source: PLoS One. 2014 Dec 2;9(12):e114042. doi: 10.1371/journal.pone.0114042 (PMC4252106; doi:10.1371/journal.pone.0114042)
Supplement: File S8 — Comprehensive discussion regarding the possibilities of calculation of atomic hydrophobicities. (DOC) [file pone.0114042.s008.doc]

# **Mapping Amphiphilicity on the Protein Molecular Surface with Atom-level Resolution**

**Dan V. Nicolau Jr^1^, Ewa Paszek^2^, Florin Fulga^2^, Dan V Nicolau^2,3^**

*^1^Department of Integrative Biology, University of California at Berkeley, Berkeley, USA*

*^2^Department of Electrical Engineering & Electronics, University of Liverpool, Liverpool, UK*

*^3^Department of Bioengineering, McGill University, Montreal, Canada*

**Supplementary Information S1**

**Discussion regarding the alternative methods for calculating atomic hydrophobicities from hydrophobicity scales**

1. **Terminology**

The following terminology (numbering system) about methods should be used:

- Methods **1, 2, and 3** refer solely to the method used to estimate the *atomic hydrophobicity* from amino acid overall hydrophobicity (i.e., as described in Chapter 3). For convenience Method 0 denominates amino acid-based hydrophobicity.
- Methods **i, ii, iii** (and more if it comes to that) refer to the way we treat the regression errors. For instance when one says that s/he calculated atomic hydrophobicity with Method1i, that means that the atomic hydrophobicity was estimated disregarding the atomic or amino acid molecular surfaces (Method 1) and for the smallest overall errors possible (Method i). These methods are described in Chapter 4.
- Methods **A, B, C** refer to the way the hydrophobicity is integrated on the molecular surface of the protein, both atomic-based and amino acid-based overall hydrophobicities.

1. **Types of Solvent Accessible Area (ASA)**

Connolly’s algorithm is geared towards the construction of the molecular surface of biomolecules, mostly proteins. To obtain this molecular surface & shape the algorithm probes the protein with virtual balls with diameters as large as the dimensions of solvent molecules, which can range, usually from 1.4A (for water) to 5A. In the process of probing the protein, the virtual ball generates three types of surfaces, which are then assigned by our PSPC the properties as follows:

- *Contact area*, when the probing ball touches only individual, single atoms. PSPC assigns the property related to the respective atom to this area.
- *Interpolated area #1*, when the probing ball touches two adjacent atoms. PSPC assigns the property related to the respective atoms to this area, pro rata.
- *Interpolated area #2*, when the probing ball touches three adjacent atoms. PSPC assigns the property related to the respective atoms to this area, pro rata.

This methodology looks appropriate for probes with small radii, but begins to loose its physical meaning when using larger probe radii, especially when assessing the hydrophobicity, which is a short range interaction. More specifically, the larger the probe radii, the larger the interpolated area at the expense of the “real” contact area. Even if the partitioning of the interpolated area is correct, the assignment of properties from atoms that are further and further away seems problematic.

1. **Estimation of atomic hydrophobicities**

We derived so far three methods to estimate atomic hydrophobicity. The first ignores the molecular surfaces of aminoacids and atoms, respectively – Method 1 (Section 3.1). The second method (Method 2, Section 3.2.) uses both areas of aminoacid and atoms in absolute terms. The third method (Method 3, Section 3.3) takes into account the ratio between molecular areas of aminoacid and atoms.

- 1. **Estimation of atomic hydrophobicities independent of exposed area**

This method (**Method 1**) calculates the atomic hydrophobicities using the following system of linear equations

For j=1 to 20; and for each AA_j_: $\sum_{i=1}^{m} hypho\_at_{i}\cdot n_{ij}=hypho\_aa_{j}$ (1)

Where j = amino acid type; i = atom type (e.g., in Bio++ there are 16types, hence m=16); AA_j_ – the j^th^ amino acid; hypho_at_i_ – atomic hydrophobicity for atom type i; n_ij_ – number of atoms of type i in aminoacid j; hypho_aa_j_ – hydrophobicity of the amino acid j.

Care should be taken to close the balance of areas, i.e., for each amino acid j, either in a peptide or in a protein, and irrespective of the probing ball, we have

$\sum_{i=1}^{m} asa\_at_{ij}\cdot n_{ij}=asa\_aa_{j}$ (2)

where asa_at_i_ – solvent accessible area of atom type i in amino acid j; n_ij_ – number of atom type i in amino acid j; and asa_aa_j_ – same for amino acid j.

*Critique*. This method assumes that hydrophobicity is a property which is independent of the surface the respective molecule and, by extension, the respective atom type, presented to the solvent (the probing ball). Therefore, hydrophobicity is an atom-additive, surface-independent property, entirely similar to the charge of the molecule or the atom.

The problem with this approach is that assigns equal weights to atoms regardless of how much area they present to the probing ball. However, all hydrophobicity scales, for amino acids, are proposing them without regard to their respective molecular surfaces, let alone as a function of the probing ball radius. The same should apply to atomic hydrophobicities.

- 1. **Estimation of the surface hydrophobicity dependent on the surface area**

An alternative method of calculation (**Method 2**) would be based on the following system of equations:

For j=1 to 20; and for each AA_j_:

$\sum_{i=1}^{m} (hypho\_at_{i}\cdot\sum_{k=1}^{l} asa\_at_{ikj})=hypho\_aa_{j}\cdot asa_{j}$ (3)

Where, in addition to the above variables, asa_jkj_ – surface presented by the k^th^ atom of type i in amino acid j; asa_j_ – surface presented by the amino acid j.

The condition for the above system (as above) is that

For j=1 to 20; and for each AA_j_:

$\sum_{i=1}^{m} \sum_{k=1}^{l} asa_{ikj}=asa_{j}$ (4)

*Critique*. This method opens many questions, possibly more problems than it can solve. For starters, hypho_aa_j_ is in most instances a quantity which is not explicitly linked to the probe radius/solvent. So, what value for asa_j_ should we use in eq. 3? Moreover, some hydrophobicities, including the most advanced, e.g. ∆G_oct_ , are related to *two* solvents (e.g. octanol and water), so what radius should we pick when calculating asa’s? Still, we should accept that hydrophobicity, e.g. ∆G_wif_, as being related to the surface of the peptide in water (which is a strong assumption, having in mind that this is related to the free enthalpy for passing a peptide through a membrane).

- 1. **Estimation of the surface hydrophobicity dependent on % of atomic asa**

An alternative method of calculation (**Method 3**) would be based on the following system of equations:

For j=1 to 20; and for each AA_j_:

$\sum_{i=1}^{m} (hypho\_at_{ij}\cdot\frac{asa\_at_{ij}}{\sum_{k=1}^{m} asa\_at_{ikj}})=hypho\_aa_{j}$ (5)

with same notations as before.

If (Eq. 4) holds, then Method 2 and Method 3 should be identical.

*Critique*. As above.

1. **Treatment of errors when estimating atomic hydrophobicities**
   1. **Minimum overall errors**

The default method (**Method i**) finds a solution to any of Eqs. 1, 3, and 5 (i.e., for Methods 1, 2 and 3, respectively), minimizing the overall error

$global\_error=\sum_{j=1}^{20} \left|hypho\_aa\_est_{j}-hypho\_aa\_actual_{j}\right|^{2}$ (6)

where hypho_aa_est_j_ is the j^th^ amino acid hydrophobicity calculated with Eqs. 1, 3 or 5, when the hypho_at have been calculated; hypho_aa_actual^j^ is the j^th^ amino acid hydrophobicity from the respective hydrophobicity scale (the default is DG_wif_).

*Critique*. While this method gives the smallest overall error, some errors for particular amino acids can be quite large. Moreover, the amino acids with larger errors could be the ones which are more frequent than amino acids with lower or no errors for estimated hydrophobicity. Nevertheless, if the mentioned problems are checked and solved, this method should give the “best” estimation of hydrophobicity on the protein surface.

- 1. **Equal errors for each amino acid**

An alternative method (**Method ii**) solves the Eqs. 1, 3, and 5 (i.e. for Methods 1, 2 and 3, respectively), minimizing the overall error (Eq. 6), but under the condition that all individual errors are equal, i.e.

$\begin{matrix} \left|hypho\_aa\_est_{1}-hypho\_aa\_actual_{1}\right|= \\ \left|hypho\_aa\_est_{2}-hypho\_aa\_actual_{2}\right|= \\ ......=\left|hypho\_aa\_est_{20}-hypho\_aa\_actual_{20}\right| \end{matrix}$ (7)

or within acceptable limits, e.g., 0.05%.

*Critique*. While this method gives equal errors for all amino acids, it might give to high overall errors. One possibility would be to relax the equalities in Eq. 7 to –say- 5% between them, instead of precise equality or too tight matching. The adoption of this method depends on the actual result of the calculation of overall errors.

- 1. **Weighted errors according to amino acid frequency**

Another alternative method (**Method iii**) solves the Eqs. 1, 3, and 5 (i.e. for Methods 1, 2 and 3, respectively), minimizing the overall error (Eq. 6), but allowing different weights for the equations in the system (Eq. 1, 3 and 5) according to a (reasonably chosen) frequency of amino acids. If each amino acid is equally probable, we actually deal with Method i above. Otherwise, the frequency of amino acids can be (a) a naturally occurring frequency, e.g. in all organisms (a1), eukaryotes (a2), prokaryotes (a3), etc. or (b) the actual frequency of amino acids on the protein studied, e.g. (b1) according to sequence of amino acids (can be taken from pdb file); (b2) according to amino acids on the surface (can be calculated with PSPC); and (b3) according to the ASA of each amino acid on the protein surface (can be calculated with PSPC).

*Critique*. This method has the potential to make results more relevant, either comparing proteins between themselves, i.e. for Method iiia; or a better calculation for one particular protein, i.e. Method iiib. This method is however a refinement, rather than something that would solve the inaccuracies if they exist with Method i.

1. **Calculation of hydrophobicity on the molecular surface of proteins**

Essentially there are two possible methods, namely using ASAs (Method A) and calculating potentials (Method B).

- 1. **Calculation of protein surface hydrophobicity using ASAs**

This approach (**Method A**) uses the amino acid-based **or** atom-based hydrophobicity and weights it with the ratio of the solvent accessible area (ASA) of the respective amino acid or atom type, respectively, on the surface of the protein. The calculation relationships are, for amino acid based hydrophobicity:

$hypho\_pr^{aa}=\sum_{j=1}^{20} hypho\_aa_{j}\cdot\sum_{n=1}^{N} \frac{asa\_aa_{nj}}{asa\_aa_{j}^{tot}}$ (8)

where hypho_pr^aa^ - protein hydrophobicity calculated with amino acid based overall hydrophobicity; asa_aa_nj_ – ASA of j^th^ amino acid in the n^th^ fragment in the protein; N – total number of amino acid j on the surface of the protein; asa_aa_j_^tot^ – total ASA of j^th^ amino acid in test tripeptides (e.g. Gly-Ala-Gly for Ala; structure optimized with Bio+).

Similarly, for atom-based hydrophobicity, we should have

$hypho\_pr^{at}=\sum_{j=1}^{20} \sum_{k=1}^{16} hypho\_at_{jk}\cdot\sum_{n=1}^{N} \frac{asa\_at_{njk}}{asa\_at_{jk}^{tot}}$ (9)

where hypho_pr^at^ - protein hydrophobicity calculated with atom-based hydrophobicity; asa_at_njk_ – ASA of of atom type k^th^ in j^th^ amino acid in the n^th^ fragment in the protein; N – total number of amino acid j on the surface of the protein; asa_at_jk_^tot^ – total ASA of the k^th^ atom type in j^th^ amino acid in test tripeptide (e.g. Gly-Ala-Gly for Ala; structure optimized with Bio+)..

*Critique*. This approach, which is –for amino acid based overall hydrophobicity- the one that is used most of the time (e.g. Eisenberg et al.) should work for atom-based hydrophobicity. For a r^2^≈0.95 and more (as we have it!); and for Eq. 4 holding, we should have similar protein hydrophobicity/hydrophilicity for both methods, within -say- a factor of 1.5-2. If we should observe something, we should have higher hydrophilicity with atom based method(s) than for aminoacid based method(s), especially when probing with larger balls. Conversely, we should have lower hydrophobicities for the same pair.

- 1. **Calculation of protein surface hydrophobicity using potentials**

This approach (**Method B**) uses the atom-based hydrophobicity as “charges” and calculates (as other proposed it, [ref]) “hydrophobic potentials” around the protein, but with an exponential, i.e. ~e^-r^ decay function rather than a hyperbolic one, i.e. ~1/r, for charges.

*Critique*. It would be entirely relevant to calculate the potentials, but the calculations appear to be very time-consuming.

- 1. **Calculation of protein surface hydrophobicity from Molecular Dynamics calculations**

This approach (**Method C**) uses the derivation of atom-based hydrophobicity from MD simulations. As above it appears as time-consuming.

*Critique*. As above.

1. **Treatment of errors when estimating atomic hydrophobicities. Minimum overall errors**

The method finds a solution to the system of the equations given by a formula (10) by minimizing the overall error according to the equation (11).

For *j*=1 to 20; and for each *AA_j_*: $\sum_{i=1}^{m} hypho\_at_{i}\cdot n_{ij}=hypho\_aa_{j}$ (10)

$global\_error=\sum_{j=1}^{20} \left|hypho\_aa\_est_{j}-hypho\_aa\_actual_{j}\right|^{2}$ (11)

Where,

*j* - amino acid type

*i* - atom type

*n_ij_* - number of atoms of type *i* in amino acid *j*

*AA_j_* - the *j^th^* amino acid

*hypho_aa_j_* - hydrophobicity of the amino acid *j*

*hypho_at_i_* - atomic hydrophobicity for atom type *i*

*hypho_aa_est_j_* - the *j^th^* amino acid hydrophobicity calculated with Eqs. 1 when *hypho_at* have been calculated

*hypho_aa_actual^j^* - the *j^th^* amino acid hydrophobicity from the respective hydrophobicity scale (the default is DG_wif_).

*Critique*. While this method gives the smallest overall error, some errors for particular amino acids can be quite large. Moreover, the amino acids with larger errors could be the ones which are more frequent than amino acids with lower or no errors for estimated hydrophobicity. Nevertheless, and if the mentioned problems are checked and accepted, this method should give the “best” estimation of hydrophobicity on protein surface.
